# Beyond prevalence: diagnostic heterogeneity in population-based glaucoma studies in Africa–a systematic review

**DOI:** 10.3389/fmed.2026.1877259

**Published:** 2026-08-04

**Authors:** Albert Kwadjo Amoah Andoh, Alexander K. Schuster, Josephine Ampong, Nana Akwasi Owusu Mensah, Kwesi Sewe, Simon Christoph König, David Ben Kumah, Kwadwo Owusu Akuffo

**Affiliations:** 1 Department of Optometry and Visual Science, College of Science, Kwame Nkrumah University of Science and Technology, Kumasi, Ghana; 2 Department of Ophthalmology, Centre for Ophthalmic Epidemiology and Healthcare Research, University Medical Centre Mainz, Mainz, Germany; 3 University of Ghana Library System, University of Ghana, Accra, Ghana

**Keywords:** Africa, diagnostic criteria, epidemiology, glaucoma, ISGEO, population-based studies, prevalence

## Abstract

**Background:**

The burden of glaucoma is disproportionately high in Africa. While numerous population-based studies have reported prevalence estimates, little attention has been given to the diagnostic criteria underpinning these findings.Objective: This review sought to systematically examine the diagnostic criteria used to define glaucoma in population-based studies in Africa, and to synthesize corresponding estimates of glaucoma prevalence.

**Methods:**

A systematic search of PubMed, Scopus, Google Scholar, and African Journals Online identified population-based studies on glaucoma prevalence in Africa. Diagnostic approaches were classified by adherence to the International Society of Geographical and Epidemiological Ophthalmology (ISGEO) criteria. The primary outcome was the diagnostic framework used, with secondary outcomes including prevalence estimates for major glaucoma subtypes.

**Results:**

Nine studies met the inclusion criteria. The use of ISGEO definitions varied considerably across the studies. Key sources of heterogeneity included intraocular pressure thresholds, optic nerve head assessment, use of visual field testing, and gonioscopy application. Studies applying the full ISGEO definitions generally had a trend of reported prevalence estimates within a narrower range (4.3% to 5.3%), whereas studies employing partial ISGEO or non-ISGEO diagnostic approaches reported a broader range of prevalence estimates (2.1% to 8.4%). The proportion of previously diagnosed glaucoma cases was low, ranging from 3.3% to 14.3% among studies that reported previously diagnosed glaucoma rates (*n* = 5).

**Conclusion:**

Diagnostic heterogeneity remains a major limitation to the comparability of glaucoma prevalence estimates across Africa. Standardized reporting of diagnostic protocols, together with clear justification of the diagnostic approaches employed, is essential to improve the interpretation and comparability of future epidemiological studies while recognizing the practical constraints of resource-limited settings.

**Systematic Review Registration:**

[https://www.crd.york.ac.uk/PROSPERO/view/ CRD42024592160], identifier [CRD42024592160].

## 1 Introduction

Glaucoma remains a major public health concern globally, estimated to affect about 111.8 million people in 2040 (1). The prevalence of glaucoma in Africa is estimated at 5.59% (2), and the region continues to bear the highest age-standardized prevalence of glaucoma-related blindness and moderate to severe visual impairment (3). Despite significant advances in diagnostic techniques and disease management, the burden of glaucoma in Africa continues to rise, driven predominantly by the high prevalence and increased age-related risk of primary open-angle glaucoma, alongside late presentation and limited access to diagnosis and treatment (4–8), as well as low levels of coverage and adherence to treatment (9, 10).

Glaucoma diagnosis relies on a combination of inherently subjective structural and functional assessments, including optic nerve head (ONH) evaluation, visual field testing, and gonioscopy (11). While in the past, intraocular pressure (IOP) measurement was part of the diagnostic criteria, nowadays this is understood as a major risk factor and not as a diagnostic criterion (12). That notwithstanding, in epidemiological studies, glaucoma diagnosis presents unique challenges because it is fundamentally based on the integration of clinical history with structural and functional ocular assessments. Although standard approaches by clinicians through comprehensive ophthalmic examinations and investigations are appropriate for epidemiological research, they are frequently unavailable in large population-based surveys conducted in many African settings. This may account for identifying probable rather than clinically confirmed glaucoma, thus, introducing the potential for diagnostic misclassification, and contributing to variability in reported prevalence estimates.

Consequently, the accurate estimation of glaucoma prevalence across population-based studies remains fundamentally constrained by the varied selection, thresholds, and completeness of these examinations reported substantially differently across prevalence studies (13). Different diagnostic criteria employed by epidemiological studies substantially influence prevalence (14), with varying diagnostic protocols estimating prevalence rates ranging from 0.4% to 11.2% (15). In response to these diversities, the International Society of Geographical and Epidemiological Ophthalmology (ISGEO) (11) discussed diagnostic definitions, leading to widely accepted criteria that improved comparability of prevalence estimates. However, subsequent evidence indicates that variable and incomplete application of these criteria persists globally (13).

Particularly in Africa, while previous reviews have provided valuable prevalence estimates in the region (2, 16–18), they have primarily emphasized disease magnitude without comprehensively examining the diagnostic approaches in defining glaucoma in population-based prevalence studies. Given that glaucoma diagnosis in epidemiological settings relies on a combination of structural and functional assessments that are variably applied, differences in case definitions represent a critical but underexplored source of variability in reported prevalence. To date, there has been limited synthesis of how the inclusion, exclusion, or modification of key diagnostic components, such as ONH evaluation, visual field testing, and gonioscopy, shapes prevalence estimates across population-based studies in the region. This gap limits the interpretability and comparability of existing epidemiological data and underscores the need for a methodologically focused synthesis that explicitly examines diagnostic criteria as a determinant of reported glaucoma burden in Africa.

This review, therefore, aims to (1) compare the diagnostic methodologies employed across studies, with particular attention to conformity with the ISGEO definitions (Table 1), and (2) summarize population-based evidence on the prevalence of glaucoma and its clinical subtypes in Africa. By consolidating these findings, the review seeks to highlight existing methodological gaps and inform the development of standardized frameworks for future epidemiological surveillance.

**TABLE 1:** The International Society of Geographical and Epidemiological Ophthalmology (ISGEO) Definition for glaucoma *(11).*

Category	Diagnostic basis	Definition
Category 1	Structural and functional evidence	Cup-to-disc ratio (CDR) or CDR asymmetry > 97.5th percentile for the normal population, or neuroretinal rim width < 0.1 CDR, with definite glaucomatous visual field defect.
Category 2	Advanced structural damage (no reliable visual field)	CDR or CDR asymmetry > 99.5th percentile when visual field testing cannot be completed.
Category 3	Structural and functional assessment not feasible	Visual acuity < 3/60 with either IOP > 99.5th percentile, evidence of glaucoma surgery, or medical records confirming glaucomatous visual morbidity.

## 2 Materials and methods

The protocol was registered in the International Prospective Register of Systematic Reviews (ID: CRD42024592160), and was conducted in accordance with the Preferred Reporting Items for Systematic Reviews and Meta-Analyses (PRISMA) guidelines (19).

### 2.1 Criteria for considering studies for this systematic review

This review synthesized evidence from population-based studies conducted across Africa that investigated the prevalence of glaucoma or its subtypes. Eligible studies included those that reported population estimates for unclassified glaucoma and its subtypes, including primary open-angle glaucoma (POAG), primary angle-closure glaucoma (PACG), secondary glaucoma (SG), or other specified and unspecified forms of the disease. Studies were included irrespective of participant age or sex, provided they used population-based sampling and reported glaucoma diagnoses based on clinical assessment or standardized criteria such as the ISGEO definitions (11). This study primarily assessed the diagnostic criteria employed in defining glaucoma. Secondary outcomes were the reported prevalence estimates for glaucoma. Eligible studies were required to determine glaucoma status through clinical examination conducted within the population-based survey using predefined diagnostic criteria. Studies reporting only self-reported glaucoma or prior clinical diagnosis without examination-based case ascertainment were not eligible for inclusion.

### 2.2 Search strategy and study selection

A comprehensive search was conducted in PubMed, Scopus, Google Scholar, and African Journals Online for studies published from inception to December 31, 2025, using the search strategy in Supplementary Table 1. These databases were chosen for their broad coverage of global and region-specific research. The search was limited to only cross-sectional population-based prevalence studies, with excluded studies encompassing non-general or hospital-based populations, unreported diagnostic criteria or glaucoma prevalence, published abstract, full article unavailable, or non-English. Retrieved studies were imported into Rayyan (20) for duplicate removal, screening, and full-text review. Two independent reviewers (AKAA and JA) screened and selected studies, and any discrepancies between reviewers were resolved through consensus, and where necessary, consultation with a third reviewer (AKS).

### 2.3 Data extraction procedure

Two reviewers (AKAA and JA) independently extracted data from each eligible study using a standardized data extraction form and subsequently cross-verified all entries for accuracy. Extracted information included the first author’s name, publication year, study country, participant characteristics (age and sex), total sample size, glaucoma subtype classification, and reported prevalence estimates. In line with the study’s objectives, detailed information on diagnostic protocols was also extracted, including IOP thresholds, criteria for ONH assessment, visual field testing, and the use of gonioscopy for angle evaluation. The degree to which each study’s diagnostic approach conformed to the ISGEO criteria was systematically documented as either full, partial, or non-ISGEO.

### 2.4 Risk of bias assessment

The Joanna Briggs Institute Prevalence Critical Appraisal Tool (JBI-PCAT) was employed in evaluating the quality of the included studies (21). The JBI-PCAT contains nine domains, which are answered with Yes, No, Unclear, and Not applicable. Based on the number of “Yes” responses, the studies were classified into three levels: high quality ( ≥ 6); moderate quality (4, 5); low quality ( ≤ 3). Two reviewers (AKAA and NAOM) assessed the quality of included studies.

### 2.5 Data synthesis

Data were synthesized using a structured narrative approach. Extracted study characteristics, diagnostic protocols, and prevalence estimates for glaucoma and its subtypes were summarized in tabular form. Diagnostic approaches were categorized based on their level of adherence to the ISGEO criteria (full, partial, or non-ISGEO) and presented in a dedicated table. Prevalence estimates were described across studies without formal quantitative pooling. Interpretation of findings considered the diagnostic frameworks used in each study; however, no direct analytical comparison between diagnostic categories and prevalence estimates was performed, given the substantial heterogeneity in diagnostic definitions, examination procedures, and study populations.

## 3 Results

### 3.1 Search results summary

**FIGURE 1:**
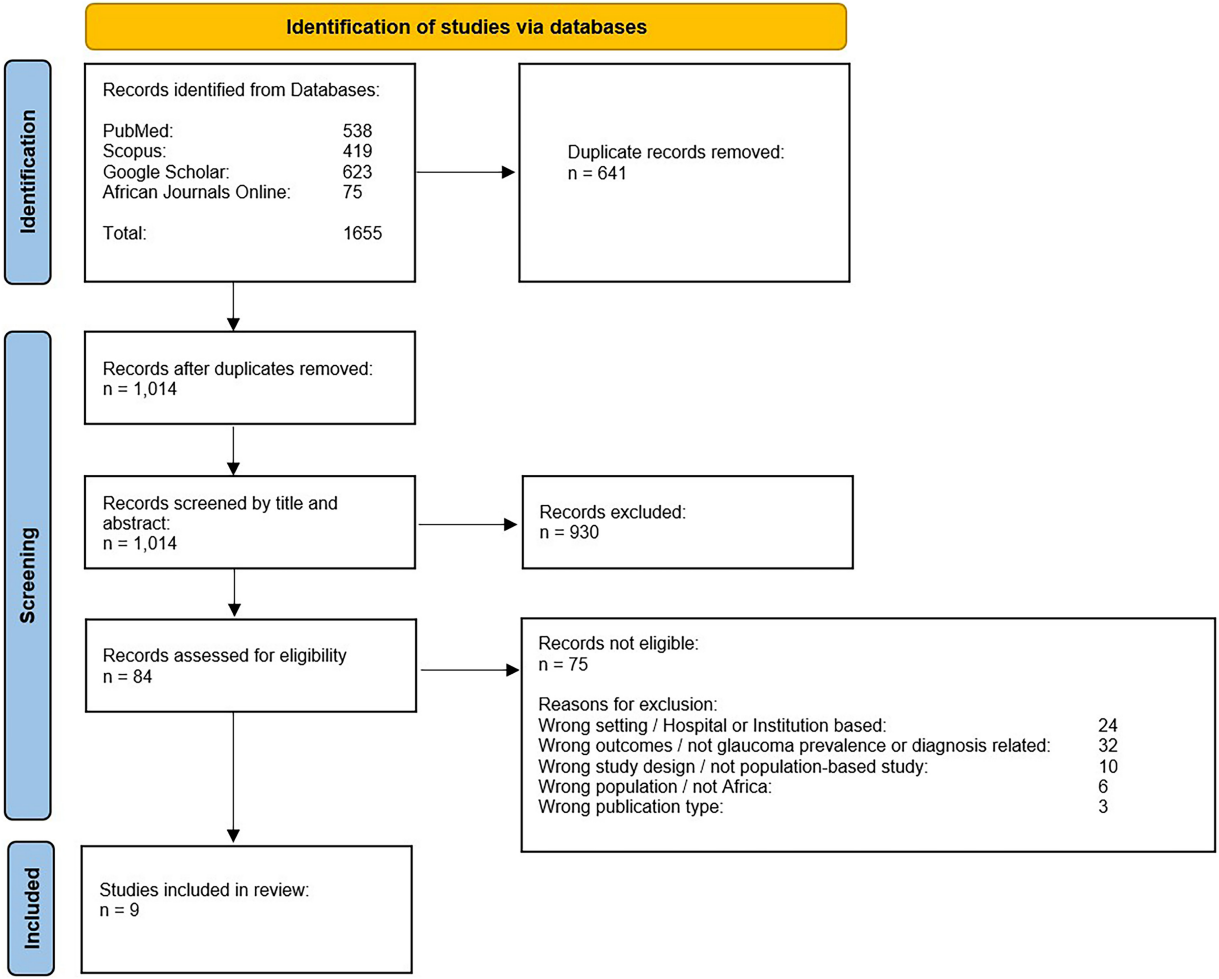
PRISMA flow diagram illustrating the study selection process for the systematic review.

**FIGURE 2:**
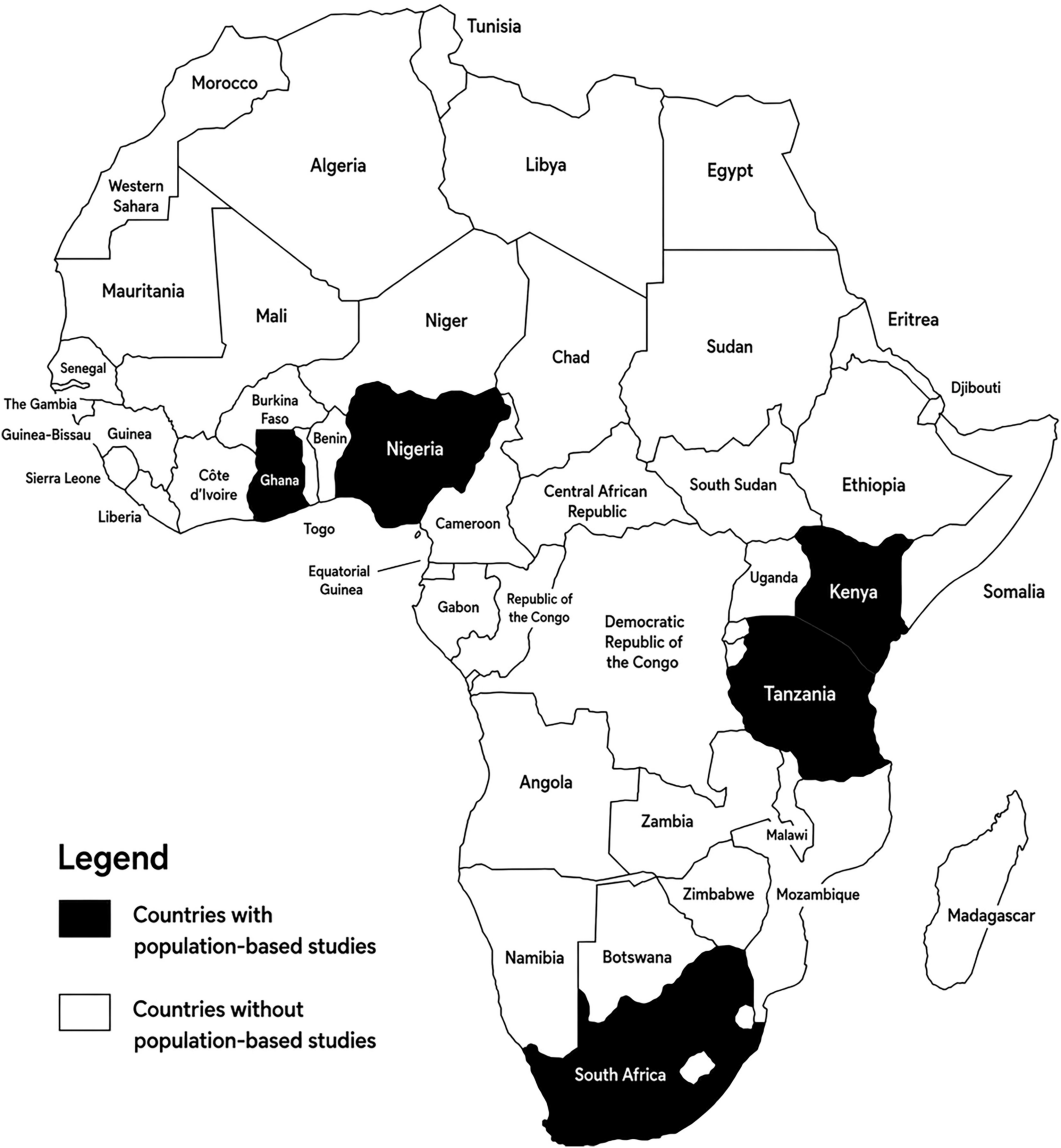
Geographic distribution of population-based studies on glaucoma prevalence across Africa.

The study selection process is summarized in Figure 1. Following database searching, duplicate removal, and screening of titles and abstracts, 84 full-text articles were assessed for eligibility. Of these, nine population-based studies met the inclusion criteria and were included in the review. These studies were published between 2000 and 2018 across five African countries, and collectively included 29,716 participants, with sample sizes ranging from 664 to 13,591 individuals. Most studies recruited adult populations, with minimum age eligibility ranging from 30 to 50 years. Geographically, the studies represented Southern Africa (*n* = 2), East Africa (*n* = 2), and West Africa (*n* = 5), shown in Figure 2. All nine studies reported the overall prevalence of glaucoma, while six studies reported prevalence estimates for POAG, and four reported PACG, in addition to other SG subtypes.

### 3.2 Diagnostic criteria across prevalence studies

#### 3.2.1 ONH assessment

The diagnostic approaches used across the included studies varied in the application of ONH assessment. ONH evaluation was consistently included across all protocols, with abnormality defined by a VCDR ≥ 0.5, or ≥ 0.7, and asymmetry thresholds between eyes ranging from > 0.1 to ≥ 0.3, or values above the 97.5th percentile for the reference population (Table 2).

**TABLE 2:** Classification of population-based glaucoma prevalence studies according to adherence to the International Society for Geographical and Epidemiological Ophthalmology (ISGEO) diagnostic criteria.

Author	Diagnostic criteria definition					Glaucoma diagnostic group		
	ONH	Visual Field Test	Gonioscopy	IOP (mmHg)	Other Parameters	Full ISGEO criteria	Partial ISGEO	Other criteria (non-ISGEO)
Buhrmann et al. (26)	C/D ≥ 0.9; or C/D > 0.7 with ≥ 1 of: abnormal RNFL, ≥ 1 clock-hour notch, or C/D asymmetry ≥ 0.3 (when disc size difference < 0.2)	Reliable glaucomatous VF defect	≥ 6 clock hours of angle closure (No view of the posterior trabecular meshwork)	> 30^s^	–			Yes
Rotchford et al. (22)	CDR ≥ 0.7 or CDR asymmetry ≥ 0.2 between eyes	Defect ≥ 12° wide (2 adjacent points), ≥ 5 dB below threshold in nerve fibre bundle pattern, confirmed on retest	Occludable = pigmented TM visible for ≤ 90° of circumference in primary position without indentation	≥ 30^s^	–	Yes		
Ekwerekwu (29)	VCDR ≥ 0.5 or asymmetry ≥ 0.2	VF defect characteristic of glaucoma	-	≥ 28^a^	–			Yes
Rotchford et al. (23)	CDR ≥ 0.7 or CDR asymmetry ≥ 0.2 between eyes.	Defect ≥ 12° wide (2 adjacent points), ≥ 5 dB below threshold in nerve fibre bundle pattern, confirmed on retest	Occludable = pigmented TM visible for ≤ 90 of circumference in primary position without indentation	≥ 30^s^	–	Yes		
Ntim-Amponsah et al. (25)	CDR ≥ 0.5 or CDR asymmetry > 0.1 between eyes	≥ 1 absolute defect within central 30°	–	> 21 or inter-eye difference >4^a^	–			Yes
Ashaye et al. (24).	VCDR ≥ 0.7 or VCDR asymmetry ≥ 0.2 or NRR width reduced to CDR ≤ 0.1	GHT outside normal limits + ≥ 3 contiguous points at 5% level on pattern deviation plot	Occludable = posterior pigmented TM visible for < 90° on gonioscopy without indentation	≥ 26^s^	-		Yes	
Budenz (28)	VCDR or asymmetry > 97.5th percentile (0.725); NRR reduced to CDR ≤ 0.1	Definite VF defect consistent with glaucoma, confirmed on retest	N/R	≥ 34^s^	–		Yes	
Kyari et al. (27)	CDR ≥ 0.7 or CDR asymmetry ≥ 0.2 between eyes	Visual field loss typical of glaucoma	Occludable = Schwalbe’s line not seen	≥ 28^s^	–	Yes		
Bastawrous et al. (30)	VCDR ≥ 0.7; asymmetry > 0.2; or abnormal features (notching, hemorrhage, pallor)	(1) Reliable test (2) GHT outside normal limits (3) ≥ 3 abnormal contiguous points in same hemifield	Occludable = pigmented TB invisible in ≥ 3/4 of angle circumference in primary position without manipulation	N/R	–	Yes		

IOP, intraocular pressure; ONH, optic nerve head; VF, visual field; CDR/VCDR, (vertical) cup-to-disc ratio; RNFL, retinal nerve fibre layer; NRR, neuroretinal rim; TM, trabecular meshwork; GHT, glaucoma hemifield test;

*^s^*IOP criteria adopted only when ONH, or field testing, or gonioscopy were not possible / cases of end-stage glaucoma (a subset of participants);

*^a^*IOP criteria were adopted for all participants; N/R, not reported.

#### 3.2.2 Visual field assessment

Visual field testing was performed in all studies, using criteria ranging from the presence of absolute or reproducible glaucomatous defects to specific thresholds of 5 dB below normal sensitivity or clusters of abnormal contiguous points on pattern deviation plots. A subset of studies used visual field confirmation to substantiate structural findings, while others incorporated visual field criteria as primary diagnostic evidence. Among the visual field assessment strategies reported, considerable variation was observed across studies. Some studies employed suprathreshold visual field screening tests, including the 66-point suprathreshold test (22), and a stepwise strategy using an initial 26-point suprathreshold test followed by 66-point and 52-point assessments (23). Others utilized the Fast 24 screening mode with confirmatory Swedish Interactive Threshold Algorithm (SITA) threshold testing (24), while one study used Frequency Doubling Technology (FDT) perimetry with the full-threshold N-30 program for functional glaucoma assessment (25).

#### 3.2.3 Gonioscopy

Gonioscopy was undertaken in six studies and applied primarily for angle classification, though the criteria for defining an occludable angle were not uniform. Buhrmann et al. (26) classified angles as occludable based on the complete absence of any view of the posterior trabecular meshwork, while Rotchford et al. (23) and Ashaye et al. (24) required that the posterior pigmented trabecular meshwork be visible for less than 90° of the angle circumference in the primary position without indentation. Also, Rotchford et al. (22) applied the more detailed Spaeth grading system, which incorporated the angle of approach, the level of iris insertion, and the iris configuration in each quadrant.

#### 3.2.4 IOP assessment

Thresholds for IOP assessment ranged from > 21 mmHg to ≥ 34 mmHg (22–29), with some studies also considering inter-eye differences greater than 4 mmHg (25). Detailed diagnostic criteria for included studies are presented in Table 2.

### 3.3 Degree of conformity of diagnostic framework to ISGEO criteria and associated prevalence estimates

Among the reviewed population-based studies, Full ISGEO criteria were defined as studies that applied the complete ISGEO diagnostic framework, including visual field assessment for all eligible participants; four studies met this definition (22, 23, 27, 30). Partial ISGEO referred to studies that followed the ISGEO framework but performed visual field assessment only in a subset of participants, typically those considered at higher risk; two studies were classified in this category (24, 28). The remaining three studies employed other diagnostic criteria (non-ISGEO), relying on study-specific definitions based on combinations of intraocular pressure thresholds, optic nerve head abnormalities, and, where applicable, visual field assessment rather than the published ISGEO framework (25, 26, 29).

Studies applying the full ISGEO criteria reported moderate but consistent glaucoma prevalence estimates ranging between 4.3% and 5.3% (22, 23, 27, 30). Partial ISGEO studies yielded slightly higher prevalence estimates, ranging from 6.5% to 7.3% (24, 28). In contrast, non-ISGEO studies demonstrated the widest variation in prevalence, from a notably lower 2.1% prevalence to 8.4% (25, 26, 29). The proportion of participants with a previous glaucoma diagnosis was reported separately from the examination-based prevalence estimates and was available in a subset of studies. Among the included studies that reported the proportion of previously diagnosed glaucoma cases in their population (*n* = 5), prior diagnosis of glaucoma was low, ranging from 3.3% to 14.3%. See Table 3.

**TABLE 3:** Comparative summary of population-based glaucoma studies in Africa and reported prevalence estimates.

Author	Country	Year	Sample size	Age group	Prevalence of glaucoma				Detection rate *n* (%)
					All glaucoma	POAG	PACG	Other forms of glaucoma	Proportion previously diagnosed
					% (95% CI)	% (95% CI)	% (95% CI)	% (95% CI)	
Rotchford et al. (22)	South Africa	2002	1,005	59.9 ± 12.1	4.5^a^ (3.2–6.1)	2.7^a^ (1.7–4.0)	0.5^c^ (0.2–1.2)	1.7^a^ (0.9–2.9) – Secondary glaucoma	5 (9.8)
Rotchford et al. (23)	South Africa	2003	839	40–97	5.3^a^ (3.9–7.1)	2.9^a^ (1.9–4.3)	0.5^a^ (0.13–1.2)	2^a^ (1.2–3.3) - Exfoliative syndrome (open-angle), Aphakic open-angle, Traumatic (angle recession), Phacolytic, and secondary angle-closure	–
Bastawrous et al. (30)	Kenya	2018	2,171	–	4.3^c^ (3.5–5.9)	–	—	–	–
Kyari et al. (27)	Nigeria	2015	13,591	≥ 40	5.02 (4.60–5.47)	–	–	–	38 (5.6)
Ashaye et al. (24)	Nigeria	2013	811	58.0 ± 12.76	7.3^c^ (5.5–9.1)	6.2^c^ (4.5–7.8)	0.2^c^ (0.0–0.6)	0.9^c^ (0.2–1.5) - Secondary glaucoma	6 (10.2)
Budenz et al. (28)	Ghana	2013	5,603	40–115	6.5 (5.8–7.1)	6.8^a^ (6.2–7.4)	–	–	12 (3.3)
Buhrmann et al. (26)	Tanzania	2000	3,247	40–99	4.16 (3.5–4.9)	3.08 (2.5–3.8)	0.59 (0.35–0.91)	Secondary OAG - 0.06 (0.01–0.22), Secondary ACG - 0.09 (0.02–0.27)	–
Ntim-Amponsah et al. (25)	Ghana	2004	1,785	≥ 30	8.4 (7.74–9.06)	8.4 (7.74–9.06)	–	–	–
Ekwerekwu (29)	Nigeria	2002	664	30+	2.1	–	–	–	2 (14.3)

*^a^*age-sex adjusted prevalence;

*^c^*Crude prevalence; CI, confidence interval; POAG, primary open-angle glaucoma; PACG, primary angle closure glaucoma; OAG, open angle glaucoma; ACG, angle closure glaucoma; *n*, frequency; %, percentage.

### 3.4 Risk of bias assessment

**FIGURE 3:**
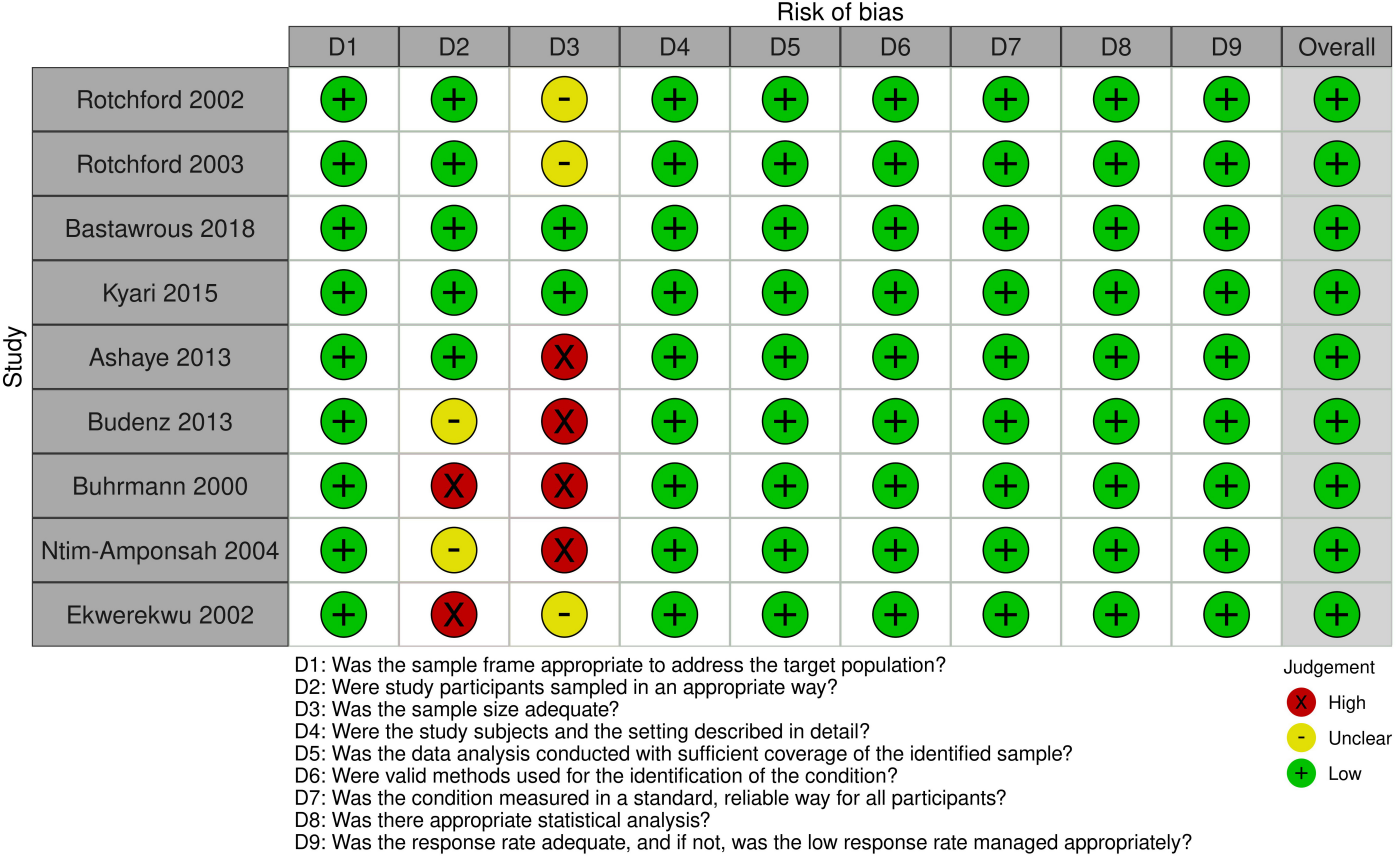
Risk of bias assessment of the included studies based on the Joanna Briggs Institute (JBI) Critical Appraisal Checklist for Prevalence Studies.

Overall, most included studies demonstrated predominantly low risk of bias across key methodological domains, including sampling strategy, sample size adequacy, participant description, and statistical analysis. Thus, all studies were considered methodologically adequate for inclusion in the review. See Figure 3.

## 4 Discussion

This review primarily highlights the substantial heterogeneity in diagnostic protocols used to define glaucoma in population-based studies conducted across Africa and how this variability coexists with wide differences in reported prevalence estimates. Across the reviewed studies, substantial heterogeneity was observed in the diagnostic protocols used to define glaucoma, criteria for ONH assessment, the use and type of visual field testing and definition of visual field defect, and the application of gonioscopy. Within this context of methodological diversity, reported glaucoma prevalence ranged from 2.1% to 8.4%, with POAG being the most frequently reported subtype.

Glaucoma can be defined as an optic neuropathy associated with characteristic structural damage to the optic nerve, usually identified along the superior and inferior poles, and an associated visual field defect, with a hemifield test grade of “outside the normal limits” and a cluster of three contiguous points at the 5% level on the pattern deviation plot (11). The diagnostic criteria for glaucoma in epidemiological research remain heterogeneous, with a comprehensive protocol typically involving multiple assessment methods such as structural assessment of the optic disc and visual field testing (13). A recent criterion proposed for defining glaucoma also emphasizes abnormality in different retinal nerve fiber layer sectors measured by optical coherence tomography, followed by a glaucoma hemifield test result, capable of achieving 85% sensitivity in detecting glaucoma (31).

Previous research has documented persistent heterogeneity in the application of standardized diagnostic frameworks across population-based prevalence studies, with about 38% of studies not using the criteria, as well as inconsistent reporting of diagnostic components such as visual field testing and gonioscopy (13). While this trend emphasizes global patterns, the present synthesis demonstrates that similar methodological variability characterizes population-based glaucoma research conducted in African settings. Importantly, the ISGEO classification published in 2002 was developed to accommodate varying levels of diagnostic information available in population-based surveys, recognizing the infeasibility of some assessments, particularly in low-resource settings. Consequently, structural-based diagnostic approaches should be viewed as contextually appropriate provided they are clearly defined and transparently reported.

Within the studies reviewed, only a subset applied the full ISGEO diagnostic criteria, while others employed partial or non-ISGEO approaches. On a global scale, a significant proportion of glaucoma epidemiological research did not use the ISGEO criteria to define glaucoma, while an increased uptake of the criteria was observed over time (13). This pattern is consistent with findings from this review, which further emphasizes that despite the introduction of ISGEO as standard glaucoma diagnostic criteria in prevalence studies, its uptake has remained low across Africa.

This low uptake of the ISGEO criteria has been reported to be due to several factors, including reliance on percentile-based thresholds without accounting for optic disc size (32–36), skewed prevalence estimates due to fixed percentile cut-offs (33, 36), overly stringent definitions for PACG that may exclude clinically relevant cases (37, 38), and not using modern imaging modalities such as the optical coherence tomography (34). Although contemporary glaucoma diagnosis increasingly incorporates optical coherence tomography, this technology was not routinely available during most included studies and remains inaccessible in many resource-limited settings across Africa. Furthermore, no internationally accepted epidemiological definition of glaucoma currently incorporates optical coherence tomography, and its integration into population-based diagnostic criteria remains an area of ongoing debate. Consequently, differences in diagnostic protocols across studies likely reflect both historical advances in diagnostic technology and resource availability, rather than methodological inconsistency alone. Prevalence estimates should therefore be interpreted within the context of the diagnostic methods available at the time each study was conducted.

Studies classified as fully conforming to ISGEO typically incorporated all levels of evidence in defining glaucoma for all subjects examined, including structural and functional evidence, advanced structural damage with unproven field loss, and cases where the optic disc was not seen or a field test was impossible. In contrast, studies categorized as partially conforming or non-ISGEO either incorporated all levels of evidence for a subset of participants or relied on a restricted set of diagnostic elements, most commonly structural optic disc assessment or elevated IOP alone, often with heterogeneous thresholds and without systematic functional confirmation. This partial implementation and selective omission of diagnostic components in African epidemiological research may be attributable to challenges such as logistical constraints, resource limitations, and feasibility concerns. Consequently, the African evidence base mirrors broader international experience in which glaucoma prevalence estimates are derived from studies with varying levels of diagnostic completeness and methodological rigor.

Harmonizing diagnostic criteria in glaucoma prevalence studies is essential, as variations in definitions directly influence reported prevalence estimates (13). Surveys that omit field testing may underestimate glaucoma prevalence, though reliance on optic nerve appearance, despite its limitations, can still capture advanced cases and yield a minimum prevalence estimate (11). While the diagnostic criteria applied in the reviewed studies lacked uniformity, studies classified as using full ISGEO definitions generally had a trend of reported prevalence estimates within a narrower range, whereas studies employing partial ISGEO or non-ISGEO diagnostic approaches reported a broader spread of prevalence values. This result aligns with the Framingham study, which showed that stricter glaucoma definitions produced lower prevalence estimates than broader criteria (15), which was also shown in data from the Rotterdam Study (14). Given the differences in diagnostic components reported, caution is warranted when interpreting and comparing reported glaucoma prevalence rates, as observed differences may reflect methodological variation rather than true differences in population-level disease burden, even when conducted in geographically or demographically similar populations.

### 4.1 Implications of the study

The findings of this review suggest that advancing glaucoma epidemiology in Africa requires a shift from broad calls for standardization toward explicit alignment between diagnostic protocols and the intended use of prevalence estimates. Although adherence to internationally recognized diagnostic frameworks, such as the ISGEO criteria, can improve comparability across epidemiological studies, the application of individual diagnostic components should be interpreted within the context of available resources and study objectives. The observed clustering of prevalence estimates within a narrower range among studies employing full ISGEO criteria, contrasted with wider variability among partial and non-ISGEO studies, indicates that diagnostic rigor materially influences the interpretability of reported prevalence. Explicit documentation of which diagnostic components were applied and how glaucoma was operationally defined would enhance secondary synthesis and reduce ambiguity in cross-study comparisons.

### 4.2 Limitations and future directions

This review has several limitations that should be considered when interpreting the findings. First, the evidence base was restricted to a relatively small number of population-based studies, reflecting the limited availability of high-quality epidemiological data on glaucoma across many African countries. Geographic representation was uneven, with no eligible population-based studies identified from some regions, particularly Central and North Africa, which may limit the generalizability of the findings to the entire continent. Second, although this review systematically categorized studies according to diagnostic protocol and conformity with international guidelines, the analysis relied on diagnostic information as reported in the original publications. Incomplete or inconsistent reporting of diagnostic methods in some studies may have led to misclassification of diagnostic rigor or underestimation of methodological variability. Third, although studies were categorized according to their conformity with the ISGEO diagnostic framework, substantial heterogeneity remained in the implementation of individual diagnostic components, including optic nerve head assessment, visual field testing, gonioscopy, and intraocular pressure thresholds. Consequently, studies classified as Full or Partial ISGEO were not methodologically identical, and differences in the application of these diagnostic components may have influenced reported prevalence estimates and limited direct comparability across studies. These findings should therefore be interpreted within the context of the diagnostic protocols employed by each study. Finally, as this review focused on cross-sectional prevalence studies, it could not account for disease progression or longitudinal diagnostic confirmation. Despite these limitations, the review provides a structured synthesis of diagnostic heterogeneity and prevalence reporting practices, offering a methodological perspective that remains essential for interpreting existing evidence and informing future research.

## 5 Conclusion

This review shows that glaucoma prevalence estimates in Africa are derived from studies with markedly heterogeneous diagnostic protocols and variable adherence to ISGEO criteria. Although prevalence consistently indicates a high burden, comparability is limited by differences in diagnostic completeness, definitions, and reporting. Highlighting diagnostic variability as a key driver of evidence gaps, this review underscores the need for greater transparency, methodological consistency, and alignment with international standards to enhance the reliability and utility of prevalence data for glaucoma control across the continent.

## Data Availability

The original contributions presented in this study are included in the article/Supplementary material, further inquiries can be directed to the corresponding authors.

## Supplementary material

Supplementary Table 1 Complete electronic search strategy for all databases included in the systematic review.

Supplementary Table 2 PRISMA 2020 checklist for the systematic review.

## References

[1] Tham YC Li X Wong TY Quigley HA Aung T Cheng CY. Global prevalence of glaucoma and projections of glaucoma burden through 2040: a systematic review and meta-analysis. *Ophthalmology*. (2014) 121:2081–90. 10.1016/j.ophtha.2014.05.013 24974815

[2] Asiamah R Kyei S Owusu G Agyiri PE. Prevalence of glaucoma in Africa: a systematic review and Bayesian meta-analysis. *PLoS One*. (2025) 20:e0330567. 10.1371/journal.pone.0330567 40811710 PMC12352844

[3] Vision Loss Expert Group of the Global Burden of Disease Study; Gbd 2019 Blindness and Vision Impairment Collaborators. Global estimates on the number of people blind or visually impaired by glaucoma: a meta-analysis from 2000 to 2020. *Eye.* (2024) 38:2036–46. 10.1038/s41433-024-02995-5 38565601 PMC11269708

[4] Abu-Amero KK González AM Osman EA Larruga JM Cabrera VM Al-Obeidan SA. Mitochondrial DNA lineages of African origin confer susceptibility to primary open-angle glaucoma in Saudi patients. *Mol Vis.* (2011) 17:1468. 21677789 PMC3110492

[5] Friedman DS Wolfs RC O’Colmain BJ Klein BE Taylor HR West S et al. Prevalence of open-angle glaucoma among adults in the United States. *Arch Ophthalmol*. (2004) 122:532–8. 10.1001/archopht.122.4.532 15078671 PMC2798086

[6] Rudnicka AR Mt-Isa S Owen CG Cook DG Ashby D. Variations in primary open-angle glaucoma prevalence by age, gender, and race: a bayesian meta-analysis. *Invest Ophthalmol Vis Sci*. (2006) 47:4254–61. 10.1167/iovs.06-0299 17003413

[7] Tielsch JM Sommer A Katz J Royall RM Quigley HA Javitt J. Racial variations in the prevalence of primary open-angle glaucoma: the baltimore eye survey. *Jama.* (1991) 266:369–74. 10.1001/jama.1991.03470030069026 2056646

[8] Abdull MM Gilbert CC Evans J. Primary open angle glaucoma in Northern Nigeria: stage at presentation and acceptance of treatment. *BMC Ophthalmol*. (2015) 15:111. 10.1186/s12886-015-0097-9 26296993 PMC4546340

[9] Mehari T Giorgis AT Shibeshi W. Level of adherence to ocular hypotensive agents and its determinant factors among glaucoma patients in menelik ii referral hospital, ethiopia. *BMC Ophthalmol*. (2016) 16:131. 10.1186/s12886-016-0316-z 27485739 PMC4969714

[10] Santos MA Ayena DK Kuaovi KR Vonor K Djagnikpo A Balo KP. [Compliance with medical treatment in primary open-angle glaucoma in Lomé]. *J Fr Ophtalmol*. (2016) 39:459–66. 10.1016/j.jfo.2015.10.013 27180648

[11] Foster PJ Buhrmann R Quigley HA Johnson GJ. The definition and classification of glaucoma in prevalence surveys. *Br J Ophthalmol*. (2002) 86:238–42. 10.1136/bjo.86.2.238 11815354 PMC1771026

[12] Loewen NA Tanna AP. *Glaucoma risk factors: Intraocular pressure*. In: Samples J Schacknow P editors. Clinical Glaucoma Care. New York, NY: Springer (2014).

[13] Al-Timimi Z Huang-Lung J Keay L Healey P Yang E Dunn HA. Systematic review of glaucoma diagnosis in prevalence studies and quality of reporting. *J Glaucoma*. (2023) 32:874–84. 10.1097/IJG.0000000000002248 37406297

[14] Wolfs RC Borger Ph Ramrattan RS Klaver CC Hulsman CA Hofman A. Changing views on open-angle glaucoma: definitions and prevalences the rotterdam study invest. *Invest Ophthalmol Vis Sci*. (2000) 41:3309–21. 11006219

[15] Kahn HA Milton RC. Alternative definitions of open-angle glaucoma. *Arch Ophthalmol*. (1980) 98:2172–7. 10.1001/archopht.1980.01020041024003 7447769

[16] Kyari F Abdull MM Bastawrous A Gilbert CE Faal H. Epidemiology of glaucoma in sub-saharan Africa: prevalence, incidence and risk factors. *Middle East Afr J Ophthalmol*. (2013) 20:111–25. 10.4103/0974-9233.110605 23741130 PMC3669488

[17] Kyari F Adekoya B Abdull MM Mohammed AS Garba F. The current status of glaucoma and glaucoma care in sub-saharan Africa. *Asia Pac J Ophthalmol*. (2018) 7:375–86. 10.22608/APO.2018392 30574693

[18] Sarfo JO Mordi P Aggrey EK Quaicoe ASP Attafuah PYA. Glaucoma prevalence and treatment in sub-saharan Africa’s elderly population: a scoping review. *BMC Geriatr*. (2025) 25:255. 10.1186/s12877-025-05901-0 40240955 PMC12004714

[19] Page MJ McKenzie JE Bossuyt PM Boutron I Hoffmann TC Mulrow CD et al. The PRISMA 2020 statement: an updated guideline for reporting systematic reviews *BMJ*. (2021) 372:n71. 10.1136/bmj.n71 33782057 PMC8005924

[20] Ouzzani M Hammady H Fedorowicz Z Elmagarmid A. Rayyan-a web and mobile app for systematic reviews. *Syst Rev*. (2016) 5:210. 10.1186/s13643-016-0384-4 27919275 PMC5139140

[21] Munn Z Tufanaru C Aromataris E. JBI’s systematic reviews: data extraction and synthesis. *Am J Nurs*. (2014) 114:49–54. 10.1097/01.NAJ.0000451683.66447.89 25742352

[22] Rotchford AP Johnson GJ. Glaucoma in zulus: a population-based cross-sectional survey in a rural district in South Africa. *Arch Ophthalmol*. (2002) 120:471–8. 10.1001/archopht.120.4.471 11934321

[23] Rotchford AP Kirwan JF Muller MA Johnson GJ Roux P. Temba glaucoma study: a population-based cross-sectional survey in urban South Africa. *Ophthalmology*. (2003) 110:376–82. 10.1016/S0161-6420(02)01568-3 12578784

[24] Ashaye A Ashaolu O Komolafe O Ajayi BG Olawoye O Olusanya B et al. Prevalence and types of glaucoma among an indigenous African population in Southwestern Nigeria. *Invest Ophthalmol Vis Sci*. (2013) 54:7410–6. 10.1167/iovs.13-12698 24135752

[25] Ntim-Amponsah CT Amoaku WM Ofosu-Amaah S Ewusi RK Idirisuriya-Khair R Nyatepe-Coo E et al. Prevalence of glaucoma in an African population. *Eye* . (2004) 18:491–7. 10.1038/sj.eye.6700674 15131680

[26] Buhrmann RR Quigley HA Barron Y West SK Oliva MS Mmbaga BB. Prevalence of glaucoma in a rural East African population. *Invest Ophthalmol Vis Sci.* (2000) 41:40–8. 10634599

[27] Kyari F Entekume G Rabiu M Spry P Wormald R Nolan W et al. A Population-based survey of the prevalence and types of glaucoma in Nigeria: results from the Nigeria national blindness and visual impairment survey. *BMC Ophthalmol*. (2015) 15:176. 10.1186/s12886-015-0160-6 26653326 PMC4676891

[28] Budenz DL Barton K Whiteside-de Vos J Schiffman J Bandi J Nolan W et al. Prevalence of glaucoma in an urban west African population: the tema eye survey. *JAMA Ophthalmol*. (2013) 131:651–8. 10.1001/jamaophthalmol.2013.1686 23538512 PMC4139110

[29] Ekwerekwu CM Umeh RE. The prevalence of glaucoma in an onchoendemic community in South-Eastern Nigeria. *West Afr J Med*. (2002) 21:200–3. 10.4314/wajm.v21i3.28029 12744567

[30] Bastawrous A Mathenge W Buchan J Kyari F Peto T Rono H et al. Glaucoma features in an East African population: a 6-year cohort study of older adults in nakuru, Kenya. *J Glaucoma*. (2018) 27:455–63. 10.1097/IJG.0000000000000941 29557831 PMC5933523

[31] Vianna JR Quach J Baek SU Quigley HA Chauhan BC. Crowd-sourced glaucoma study. *Am J Ophthalmol*. (2025) 280:283–97. 10.1016/j.ajo.2025.08.016 40816664

[32] Vijaya L George R Baskaran M Arvind H Raju P Ramesh SV et al. Prevalence of primary open-angle glaucoma in an urban south Indian population and comparison with a rural population. the chennai glaucoma study. *Ophthalmology.* (2008) 115:648e–54e. 10.1016/j.ophtha.2007.04.062 17664010

[33] Nangia V Jonas JB Matin A Bhojwani K Sinha A Kulkarni M et al. Prevalence and associated factors of glaucoma in rural central India. *PLoS One*. (2013) 8:e76434. 10.1371/journal.pone.0076434 24098790 PMC3787001

[34] McCann P Hogg R Wright DM Pose-Bazarra S Chakravarthy U Peto T et al. Glaucoma in the northern Ireland cohort for the longitudinal study of ageing (NICOLA): cohort profile, prevalence, awareness and associations. *Br J Ophthalmol*. (2020) 104:1492–9. 10.1136/bjophthalmol-2019-315330 32034006

[35] Sakata K Sakata LM Sakata VM Santini C Hopker LM Bernardes R et al. Prevalence of glaucoma in a south Brazilian population: projeto glaucoma. *Invest Ophthalmol Vis Sci*. (2007) 48:4974–9. 10.1167/iovs.07-0342 17962447

[36] Liang YB Friedman DS Zhou Q Yang X Sun LP Guo LX et al. Prevalence of primary open angle glaucoma in a rural adult Chinese population: the handan eye study. *Invest Ophthalmol Vis Sci*. (2011) 52:8250–7. 10.1167/iovs.11-7472 21896871

[37] Foster PJ Aung T Nolan WP Machin D Baasanhu J Khaw PT et al. Defining “occludable”; angles in population surveys: drainage angle width, peripheral anterior synechiae, and glaucomatous optic neuropathy in east Asian people. *Br J Ophthalmol*. (2004) 88:486–90. 10.1136/bjo.2003.020016 15031161 PMC1772081

[38] Casson RJ Baker M Edussuriya K Senaratne T Selva D Sennanayake S. prevalence and determinants of angle closure in central Sri Lanka: the kandy eye study. *Ophthalmology.* (2009) 116:1444–9. 10.1016/j.ophtha.2009.03.005 19559485

