# Supplementary material for: Beyond prevalence: diagnostic heterogeneity in population-based glaucoma studies in Africa–a systematic review
Source: Front Med (Lausanne). 2026 Aug 4;13:1877259. doi: 10.3389/fmed.2026.1877259 (PMC13487683; doi:10.3389/fmed.2026.1877259)
Supplement: Supplementary file 1 [file Table_1.doc]

**Search Strategy**

| **Database** |  | **Date** | **Search Strategy** |
| --- | --- | --- | --- |
|  | **Pubmed** |  | 1. (glaucoma[MeSH Terms] OR glaucoma*[Title/Abstract] OR "primary open angle glaucoma"[Title/Abstract] OR "primary angle closure glaucoma"[Title/Abstract] OR "secondary open angle glaucoma"[Title/Abstract] OR "congenital glaucoma"[Title/Abstract]) 2. (prevalence[MeSH Terms] OR epidemiology[Subheading] OR prevalence[Title/Abstract] OR epidemiolog*[Title/Abstract] OR "population-based"[Title/Abstract]) 3. (diagnosis[MeSH Terms] OR diagnos*[Title/Abstract] OR "diagnostic criteria"[Title/Abstract] OR "case definition*"[Title/Abstract] OR ISGEO[Title/Abstract]) 4. (Africa[MeSH Terms] OR africa*[Title/Abstract] OR "sub saharan africa"[Title/Abstract] OR "north africa"[Title/Abstract] OR "west africa"[Title/Abstract] OR "east africa"[Title/Abstract] OR "southern africa"[Title/Abstract]) 5. 1# AND #2 AND #3 AND #4 |
|  |  |  |  |
|  |  |  |  |
|  |  |  |  |
|  |  |  |  |
| **Google Scholar** |  |  | Search ID \| Search string used \| Date searched \| Sort order \| Results screened \| Results retained \| Notes; GS-1 \| "glaucoma" "prevalence" Africa diagnosis \| DD Month YYYY \| Relevance \| First 200 \| n = ___ \| Core prevalence search; GS-2 \| "glaucoma prevalence" Africa "diagnostic criteria" \| DD Month YYYY \| Relevance \| First 200 \| n = ___ \| Diagnostic emphasis; GS-3 \| "glaucoma" Africa ISGEO prevalence \| DD Month YYYY \| Relevance \| First 200 \| n = ___ \| ISGEO-specific; GS-4 \| "population-based" glaucoma Africa prevalence \| DD Month YYYY \| Relevance \| First 200 \| n = ___ \| Study design focus |
| **Scopus** |  |  | 1. TITLE-ABS-KEY ( glaucoma* OR "open angle glaucoma" OR "angle closure glaucoma" OR "primary open angle glaucoma" OR POAG ) 2. TITLE-ABS-KEY ( prevalence OR epidemiolog* OR burden OR "population-based" OR "community-based" ) 3. TITLE-ABS-KEY ( diagnos* OR "diagnostic criteria" OR "case definition*" OR screening OR ISGEO OR "International Society of Geographical and Epidemiological Ophthalmology") 4. TITLE-ABS-KEY ( africa* OR "sub saharan africa" OR "West Africa" OR "North Africa" OR "East Africa" OR "Central Africa " OR "southern Africa" OR algeria OR angola OR benin OR botswana OR "burkina faso" OR Burundi OR "cape verde" OR "cabo verde" OR cameroon OR chad OR "central african republic" OR comoros OR congo OR "democratic republic of congo" OR djibouti OR egypt OR eritrea OR eswatini OR Swaziland OR ethiopia OR gabon OR gambia OR ghana OR guinea OR "guinea bissau" OR "cote d'ivoire" OR "ivory coast" OR kenya OR lesotho OR liberia OR Libya OR madagascar OR malawi OR mali OR mauritania OR mauritius OR Morocco OR mozambique OR namibia OR niger OR Nigeria OR rwanda OR senegal OR seychelles OR "sierra leone" OR somalia OR "south africa" OR "south sudan" OR sudan OR tanzania OR togo OR tunisia OR uganda OR zambia OR zimbabwe ) 5. #1 AND #2 AND #3 AND #4 |
| **African Journals Online** |  |  | AJOL Search Strings:  AJOL-1 (Primary search – prevalence focus)  **glaucoma prevalence**  AJOL-2 (Secondary search – diagnostic focus)  **glaucoma diagnosis**  AJOL-3 (Secondary search – study design focus)  **glaucoma population-based**  AJOL-4 (Secondary search – geographic focus)  **glaucoma Africa**  AJOL-5 (Optional – diagnostic criteria)  **glaucoma diagnostic criteria** |
